# Comparative clinical evaluation of breast augmentation using silicone foam coated implants and textured implants^1^

**DOI:** 10.1590/s0102-865020200040000007

**Published:** 2020-06-12

**Authors:** Ivana Leme de Calaes, Marcos Matias Motta, Rafael de Campos Basso, Davi Reis Calderoni, Paulo Kharmandayan

**Affiliations:** I Fellow PhD degree, Postgraduate Program in Surgical Sciences, Faculty of Medical Sciences, Universidade Estadual de Campinas (UNICAMP), Brazil. Scientific, intellectual, conception and design of the study; acquisition, analysis and interpretation of data; technical procedures; statistics analysis; manuscript preparation and writing; final approval.; II Assistant Professor, Division of Plastic Surgery, UNICAMP, Campinas-SP, Brazil. Scientific and intellectual content of the study, technical procedures.; III Assistant Professor, Division of Plastic Surgery, UNICAMP, Campinas-SP, Brazil. Scientific and intellectual content of the study, statistics analysis.; IV PhD, Division of Plastic Surgery, UNICAMP, Campinas-SP, Brazil. Scientific, intellectual, conception and design of the study; technical procedures; manuscript preparation.; V Full Professor and Chairman, Division of Plastic Surgery, UNICAMP, Campinas-SP, Brazil. Scientific, intellectual, conception and design of the study; analysis and interpretation of data; technical procedures; manuscript preparation; critical revision; final approval.

**Keywords:** Silicone Elastomers, Prostheses and Implants, Breast Implants, Mammaplasty

## Abstract

**Purpose:**

To evaluate whether silicone foam implants have a different evolution pattern compared to conventional texture implants.

**Methods:**

Fifty-eight female patients underwent surgery. They were divided into two groups (silicone foam – Lifesil® – and microtexturized silicone – Lifesil®). The evolution was analyzed in postoperative consultations, with physical examination, photographic documentation and filling in a satisfaction questionnaire, in the postoperative period of one month, four months, one year and then annually, up to a maximum of 3 years of follow-up.

**Results:**

There were no statistically significant differences in presence of rippling, stretch marks, breast ptosis, capsular contracture and quality of scars. There was a higher rate of patients who were very satisfied with the outcome 360 days after surgery in the group receiving silicone foam implants (p = 0.036).

**Conclusion:**

In short time, silicone foam envelope implants proved to be as reliable as textured silicone envelope implants, making them an option for augmentation mammoplasty.

## Introduction

Breasts have always occupied a prominent place in female body aesthetics, acting on sensuality and improving women’s self-esteem. In the past, small breasts were the desire of the majority, but in recent decades there has been a change in the concept of beauty in this body segment, with women with larger breasts and a more marked upper pole, due to the use of implants in the media. Therefore, breast augmentation is the most performed plastic surgery in Brazil today, reaching 197.577 procedures in 2018, according to the Brazilian Society of Plastic Surgery (18.8% of cosmetic surgeries performed). There is also an increasing number of surgeries for breast fat grafting, helping to improve body contouring, in addition to the possibility of obtaining more favorable aesthetic results in cases of breast reconstruction after cancer and other thoracic deformities.

Throughout history, different materials have been used for breast augmentation, from paraffin injections to the current use of silicone implants. The first silicone gel breast implant was developed by Cronin and Gerow in 1963, giving rise to a number of changes, mainly in relation to the surface, characterized primarily by its smooth texture. Although these innovations increased the degree of satisfaction compared to previous results, there was still a very common complication: capsular contracture.

Histologically, the capsule has several layers composed of fibroblasts, fibrocytes, myofibroblasts and histiocytes surrounded by acellular tissue, rich in collagen fibers. This layer, in circular arrangement, exerts traction on the implant and determines signs and symptoms ranging from discomfort or breast stiffening sensation to continuous and refractory pain, with possible loss of mobility and aesthetic deformity^1^. Capsular contracture may occur at any moment after surgery and this condition is diagnosed clinically, as mentioned above, but imaging tests are usually requested for diagnostic confirmation^2^.

Different factors may determine the formation and intensity of the peri-implant capsule: hematoma, subclinical infection, silicone extravasation, type of coating, plan of the prosthesis’ placement, and immune response^3^. It has been suggested that chronic inflammation plays a fundamental role in the etiopathogenesis of capsular contracture, with several inflammatory mediators already identified, such as Interleukins 1 and 6, TNF-alpha, platelet-derived growth factors and TGF-beta. The presence of a large number of macrophages, multinucleated giant cells and myofibroblasts favor the inflammatory mechanism as a key factor in the development of capsular contracture, also generating the expression of high levels of the type II leukotriene receptor and pro cytokine mRNA. -inflammatory TNF-alpha in severely contracted capsules. The presence of myofibroblasts in the contracted capsule has been reported to produce smooth muscle alpha actin (alpha-SMA), where the most severely deformed capsules have a higher production of alpha-SMA, suggesting a direct role for activated myofibroblasts in the development of contracture^4^. In addition, more recent studies on the cellular composition of intracapsular lymphocytes suggest a TH1/TH17-weighted local immune response, ensuing fibrosis is promoted by the production of profibrotic cytokines as a consequence of faltering function of local T regulatory cells^5,6^.

The coating of the implant was proven to be an important factor for major reduction in the formation of capsular contracture, leading most breast implant manufactures to replace the production of smooth coating with that of polyurethane-coated implants or textured implants, because the reduction of the capsular contracture index was attributed to the three-dimensional structure obtained with this configuration, which causes the vector sum resulting from the contraction of capsular fibers to be smaller. In the 90s, implants also started to be produced with a more cohesive type of silicone gel and multilayer wrap (in addition to the textured surface), with the aim of reducing silicone leakage (bleeding) and, thereby, reducing the complication rate (in addition to reducing capsular contracture, it is now known that less inflammatory activity around implants can decrease the risk of autoimmune diseases related to these devices); the mechanisms driving aberrant immune responses are mostly unknown and deserve further study, but a genetic predisposition has been found to play a pivotal role in the development of autoimmunity^6^.

**Figure 1:**
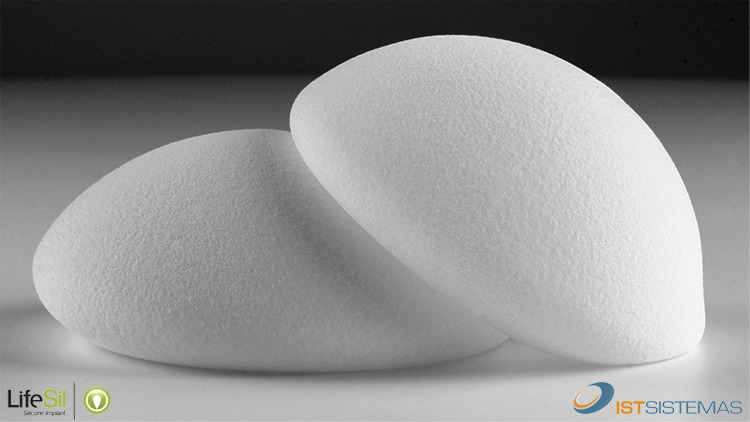
Silicone foam coated implants.

On the other hand, the use of silicone implants coated with polyurethane has been target of several criticisms due to the difficulty of removal in a possible surgical retreatment, as well as to questions about the toxicity caused by the metabolism of the material in the long term, and also to the uncertainties regarding possible damages to the organism caused by the intense inflammatory reaction produced. Thus, Wagenführ Jr^7^ developed silicone implants with a high degree of cohesiveness inside, with multiple layer wrapping, as well as external coating with silicone foam (Fig. 1), which would theoretically have the advantage of the foam’s structure being similar to that of polyurethane without, however, featuring negative aspects such as degradation, inflammation and possible toxicity of catabolites such as 2-4 TDA (toluenediamine).

**Figure 2:**
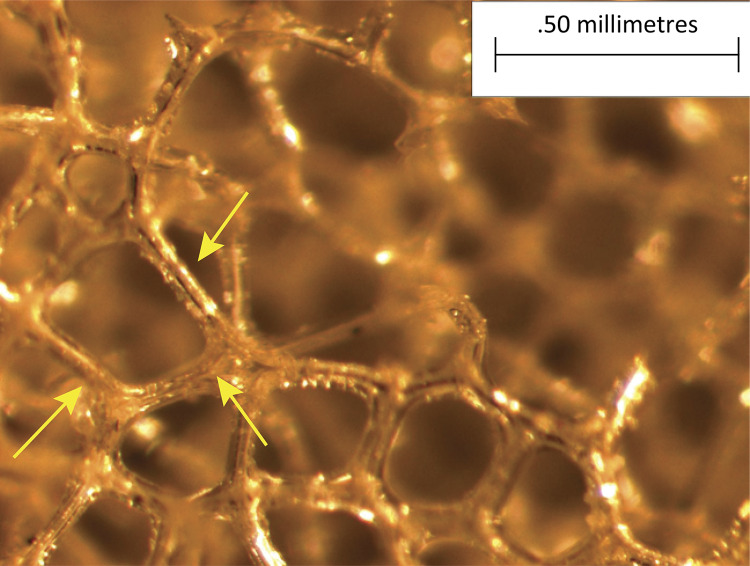
Photomicrography of polyurethane foam implant. Implant with polyurethane foam increased by x57. Arrows pointing the polyurethane beams.

**Figure 3:**
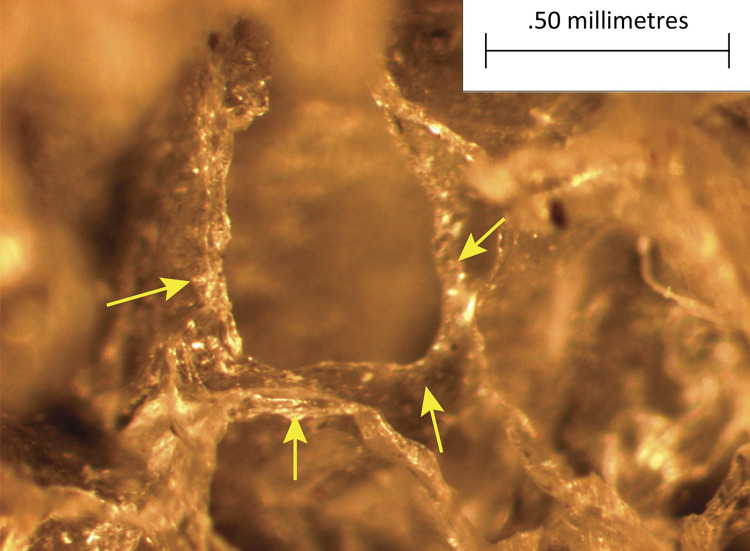
Photomicrograph of silicone foam implant. Implant with silicone foam increased by x57. Arrows pointing the silicone walls.

**Figure 4:**
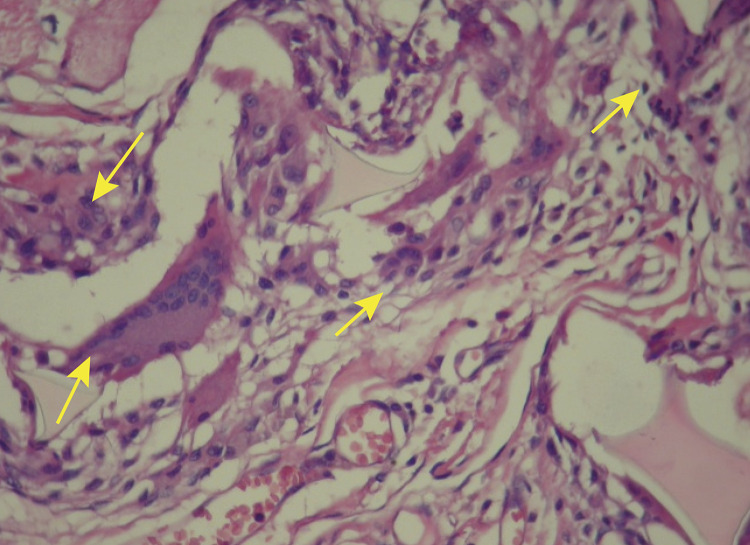
Photomicrography of polyurethane foam with intense strange body reaction. Polyurethane group animal (HE, x200). Arrows indicating the large number of giant cells.

**Figure 5:**
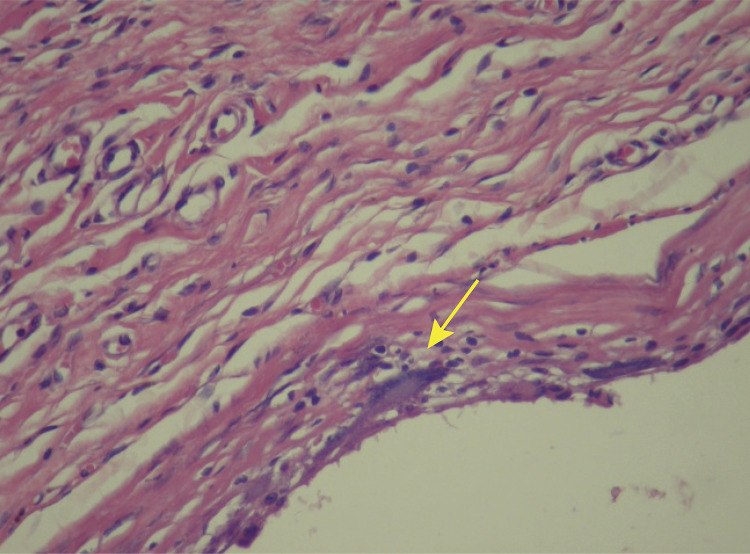
Photomicrography of silicone foam with discreet strange body reaction. Silicone group animal (HE, x200). Arrow indicating a giant cell.

In Wagenführ’s Jr^7^ study, comparing disks coated with polyurethane foam and disks coated with silicone foam (Figs. 2 and 3), he found that the foreign body reaction was moderate or intense (Fig. 4) for all animals in the polyurethane group and in every moment. In the explanted capsules of the rats that received discs coated with silicone foam, there was absence or slight presence of the reaction (Fig. 5), with a significant difference between the groups. Thus, it is concluded in that study that the silicone foam could have a greater biocompatibility than the polyurethane foam, given the differences found in the foreign body reaction process.

In 2009, Balderrama^8^ confirmed this expectation through experimental studies. Among these issues, the following can be mentioned: greater adhesion of this type of coating (silicone foam) to the breast tissues, resulting in a lower rate of breast ptosis in the late postoperative period, and less visualization of rippling, especially in the upper pole of the breasts of slim patients, as Wagenführ Jr^7^ supposed.

This prospective study was designed to clarify the clinical evolution of this new material for coating the implants (silicone foam), when compared to the textured silicone implants, given that there are still no studies published in the literature with this new type of material, and also evaluated possible differences in the patients’ complaints, such as: incidence of capsular contracture, stretch marks (due to tissue distension resulting from the inclusion of the implants), scar quality and patient satisfaction rate after surgery.

## Methods

Prospective randomized clinical trial (after approval by the UNICAMP Ethics Committee). 60 patients with a minimum age of 18 years old and complaints of hypomastia were selected at the UNICAMP Clinical Hospital, whose inclusion criteria were an indication of breast augmentation with implants silicone. The minimum number of patients in the sample was determined by the university’s statistics department. Patients who had underwent previous inclusion of breast implants, patients with mastectomy sequelae, pregnant women, patients with mammary ptosis (who required mastopexy), and those with breast abnormalities or nodules with BI-RADS classification 4 or higher in the preoperative ultrasonography and/or mammography were excluded.

For inclusion in the research protocol, the patients had to sign the Informed Consent Form. Preoperative examinations were requested (complete blood count, coagulogram, urea, creatinine, blood glucose, electrocardiogram, chest X-ray, breast ultrasound and mammography) and the breasts were measured to determine the volume of the implants. The latter were provided by the Lifesil Silicone Implant company, in two models: silicone foam coated and textured surface (microtexture texture). The models were sent on the day of surgery for randomization only in the intraoperative period. The patients had no surgery expenditure. Both models of implants are released for commercialization by ANVISA, health regulatory agency in Brazil.

An incision (4 cm) in the inframammary sulcus and dissection in the subglandular plane were standardized; then, the coating of the implants (always bilateral) was randomly chosen. Randomization was performed in a block, using ten closed opaque envelopes (brown paper) containing sequential numbers from 1 to 10; the anesthetist or room circulator would draw one of these envelopes, and thus the number contained within the envelopes was matched with matching tables (6 tables containing the sequence from 1 to 10, with the type of coating that number corresponded to, then totaling types of implants that the 60 patients would receive). The use of six tables (identified as tables A, B, C, D, E and F), numbered from 1 to 10 (and not a single table numbered from 1 to 60) provided greater security for the termination of the search, since if the work was terminated early for any reason, we would have a more even number between the patients in groups A and B (each table contained 5 numbers corresponding to silicone foam implants and 5 numbers corresponding to textured implants). In this way, the patients were randomly assigned to two groups with 30 individuals each: Group A (silicone foam coated) and Group B (textured silicone).

The clinical evaluations were performed by the author and/or supervisor of the study, 30 days, 120 days, 360 days after surgery, and then annually; resident physicians did not perform care or measurements of patients in this study. Patient complaints were recorded and quality of scars, presence of stretch marks, rippling, breast ptosis and possible presence of capsular contracture were evaluated (according to Baker’s classification).

The evaluation could not be blind, as the only two evaluators were also surgeons. The implants are macroscopically different (the silicone foam is more opaque, whitish), and there is often the memory of which implant we had inserted in that patient, even without looking at the chart, making blind analysis impossible. Although the implants were donated by the company Lifesil, the analyses were not interfered by the sponsor.

Standardized photographic documentation (front-on, semi-profile and profile portraits), captured by a Canon G7 camera, was obtained during the different evaluation periods. After each visit, the patients filled out a questionnaire in which they scored from 0 to 10 personal satisfaction on the following questions: stretch marks, scar, ptosis (sagging) of the breasts and general satisfaction with the result. There was a table to standardize the score: 0-2 (very dissatisfied), 3-4 (unsatisfied), 5-6 (indifferent), 7-8 (satisfied) and 9-10 (very satisfied).

A minimum period of 01 year of postoperative follow-up was expected for data submission to statistical analysis. To compare categorical variables, the chi-square test was used and, when necessary, Fisher’s exact test. For comparison of numerical variables, the Mann-Whitney test was used. In this statistical analysis, the following software were used: SPSS V20, Minitab 16 and Excel Office 2010. 95% confidence interval was established (p <0.05).

## Results

The surgeries were performed between July 2014 and March 2017; in this period, two patients were excluded, one belonging to group A and the other to group B, for different reasons: pregnancy and loss of follow-up, respectively. Thus, the statistical analysis was held with n=58 patients. Analysis consists of 58 patients whose postoperative minimal follow-up period was 1 year, 38 patients whose postoperative minimal follow-up period was 2 years, and 16 patients whose postoperative follow-up period was 3 years.

The mean age for Group A was 22.86 years old (19-39 years) and 23.86 years old for Group B (19-50 years). There was no significant difference between groups (p=0.3063).

About the ethnicity of the patients, we have 23 Caucasian patients, 04 oriental patients and 02 multiethnic patients in group A. In group B, we have 25 Caucasian patients, 02 oriental patients and 02 afro-descendant patients. There was no significant difference between groups (p=0,183), considering Caucasians and non-Caucasians.

In the comparison of groups to the implants’ volume (Table 1), there was statistical difference between the groups: Group A (silicone foam) had a higher mean than Group B (p-value<0.05 in the 3 comparisons):

**Table 1:** Implants volume.

Implants volume		Mean	Median	N	P-value
Right	Group A	287.9	275	29	0.013
	Group B	264.7	250	29	
Left	Group A	292.2	275	29	0.002
	Group B	258.6	250	29	
Medium	Group A	290.1	275	29	0.005
	Group B	261.6	250	29	

**Figure 6:**
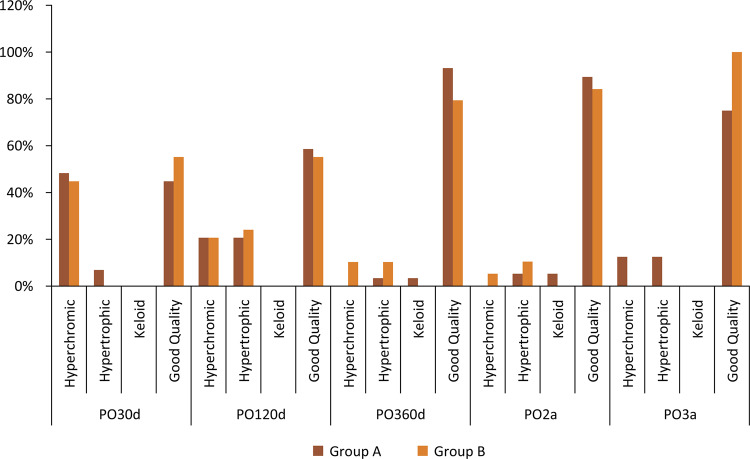
Scar comparison.

Scars were classified as great in the majority of patients with 1 year or more of postoperative follow-up; only one patient had keloid (Caucasian patient). The differences between the groups were not significant (Fig. 6 and Table 2).

**Table 2:** Scar comparison.

Scar		Group A		Group B		Total		P-value
		N	%	N	%	N	%	
PO30d	Hyperchromic	14	48.3%	13	44.8%	27	46.6%	0.376
	Hypertrophic	2	6.9%	0	0.0%	2	3.4%	
	Keloid	0	0.0%	0	0.0%	0	0.0%	
	Good Quality	13	44.8%	16	55.2%	29	50%	
PO120d	Hyperchromic	6	20.7%	6	20.7%	12	20.7%	0.732
	Hypertrophic	6	20.7%	7	24.1%	13	22.4%	
	Keloid	0	0.0%	0	0.0%	0	0.0%	
	Good Quality	17	58.6%	16	55.2%	33	56.9%	
PO360d	Hyperchromic	0	0.0%	3	10.3%	3	5.2%	0.097
	Hypertrophic	1	3.4%	3	10.3%	4	6.9%	
	Keloid	1	3.4%	0	0.0%	1	1.7%	
	Good Quality	27	93.2%	23	79.4%	50	86.2%	
PO2y	Hyperchromic	0	0.0%	1	5.3%	1	2.6%	0.615
	Hypertrophic	1	5.3%	2	10.5%	3	7.9%	
	Keloid	1	5.3%	0	0.0%	1	2.6%	
	Good Quality	17	89.4%	16	84.2%	33	86.9%	
PO3y	Hyperchromic	1	12.5%	0	0.0%	1	6.3%	0.076
	Hypertrophic	1	12.5%	0	0.0%	1	6.3%	
	Keloid	0	0.0%	0	0.0%	0	0.0%	
	Good Quality	6	75.0%	8	100.0%	14	87.4%	

In relation to capsular contracture, no patient in group A had this complication. Two patients in group B (fine texture) presented capsular contracture at the end of the first year of follow-up, a total of 6.9% patients in this group (3.45% of the total sample of operated patients). Incidences were divided according to location (Table 3).

**Table 3:** Breast contracture comparison.

Breast contracture		Group A		Group B		Total		P-value
		N	%	N	%	N	%	
30 days of postoperative follow-up	Absent	29	100%	29	100%	58	100%	- × -
120 days of postoperative follow-up	Absent	29	100%	28	96.6%	57	98.3%	0.313
	Baker 2	0	0.0%	1	3.4%	1	1.7%	
360 days of postoperative follow-up	Absent	29	100%	27	93.1%	56	96.6%	0.150
	Baker 2	0	0.0%	2	6.9%	2	3.4%	
2 years of postoperative follow-up	Absent	19	100%	17	89.5%	36	94.7%	0.146
	Baker 2	0	0.0%	2	10.5%	2	5.3%	
3 years of postoperative follow-up	Absent	8	100%	7	87.5%	15	93.8%	0.302
	Baker 3 & 4	0	0.0%	1	12.5%	1	6.3%	

Despite being present only in group B, capsular contracture had no statistically significant difference between groups.

**Figure 7:**
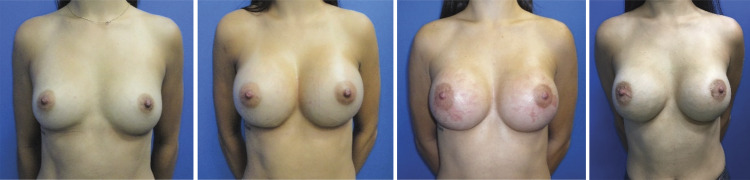
Preoperative, PO 1m, PO 4m (with bilateral stretch marks) and PO 1 year, with improvement of the stretch marks.

The presence of new striae was evaluated in the different periods (Fig. 7). There was no statistical difference (p=0.319).

Satisfaction rate (very dissatisfied, dissatisfied, indifferent, satisfied and very satisfied) was also analyzed (Table 4); there were no very dissatisfied patients. The 360-days postoperative follow-up showed a higher incidence of patients who were very satisfied with the result in group A (silicone foam), when compared to group B, with statistical significance (p-value=0.036).

**Table 4:** Satisfaction scores comparison.

Satisfaction Scores		Group A		Group B		Total		P-value
		N	%	N	%	N	%	
PO30d	Dissatisfied	0	0.0%	1	3.4%	1	1.7%	0.209
	Satisfied	3	10.3%	7	24.1%	10	17.2%	
	Very Satisfied	26	89.7%	21	72.4%	47	81.0%	
PO120d	Dissatisfied	1	3.4%	1	3.4%	2	3.4%	0.252
	Indifferent	0	0.0%	1	3.4%	1	1.7%	
	Satisfied	3	10.3%	8	27.6%	11	19.0%	
	Very Satisfied	25	86.2%	19	65.5%	44	75.9%	
PO360d	Satisfied	4	13.8%	11	37.9%	15	25.9%	0.036
	Very Satisfied	25	86.2%	18	62.1%	43	74.1%	
PO2y	Satisfied	3	15.8%	8	42.1%	11	28.9%	0.074
	Very Satisfied	16	84.2%	11	57.9%	27	71.1%	
PO3y	Satisfied	2	25.0%	3	37.5%	5	31.3%	0.590
	Very Satisfied	6	75.0%	5	62.5%	11	68.8%	

The sternal furcula-nipple distance was measured and we considered the Regnault classification to evaluate breast ptosis (Table 5): no significant difference in ptosis over time for both groups:

**Table 5:** Breast ptosis comparison.

Ptosis		Group A		Group B		Total		P-value
		N	%	N	%	N	%	
PO30d	Absent	29	100%	29	100%	58	100%	- x -
PO120d	Absent	28	96.6%	26	8.,7%	54	93.1%	0.300
	Pseudoptosis	1	3.4%	3	10.3%	4	6.9%	
PO360d	Absent	27	93.1%	27	93.1%	54	93.1%	0.513
	Grade I	1	3.4%	0	0.0%	1	1.7%	
	Pseudoptosis	1	3.4%	2	6.9%	3	5.2%	
PO2y	Absent	18	94.7%	19	100%	37	97.4%	0.311
	Pseudoptosis	1	5.3%	0	0.0%	1	2.6%	
PO3y	Absent	8	100%	8	100%	16	100%	- x -

**Figure 8:**
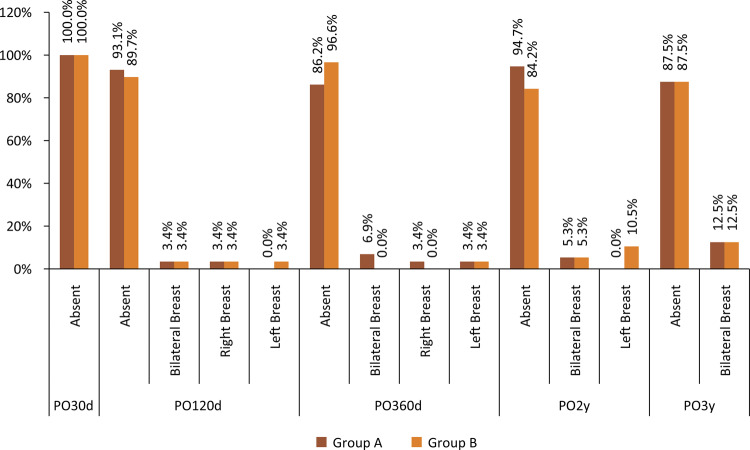
Rippling comparison.

**Figure 9:**
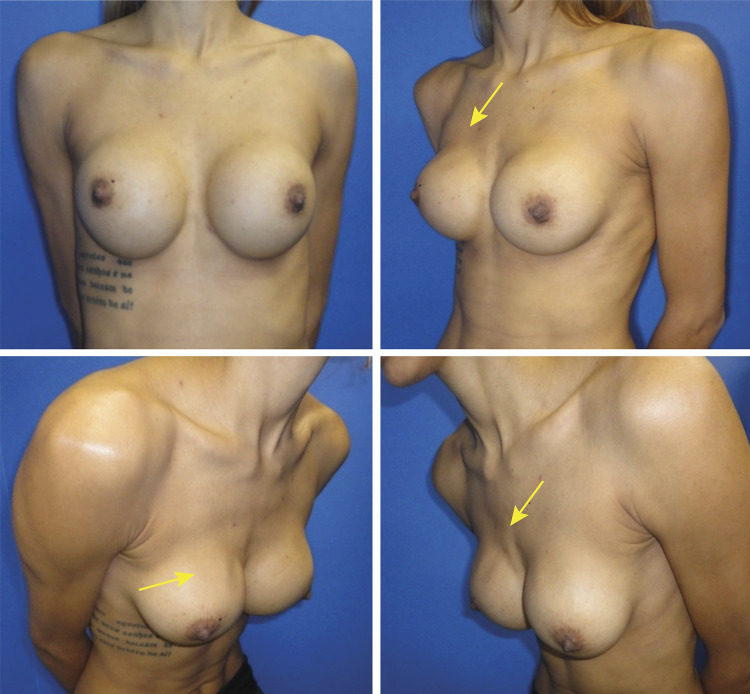
FFFB patient (Group B), in PO 2 years, with weight loss and a wavy appearance in the super-medial section of the right breast, is not visible in photo 10, but it is evident in the other photos, according to the arrows.

Regarding the presence of rippling (Figs. 8 and 9), there was also no significant difference between the groups (p>0.05 all the times).

## Discussion

The characteristics of an ideal implant were defined by Scales^9^ in 1953. The Silastic® prosthesis was produced with the purpose of solving the problem of silicone leakage through the implant’s wall and of detachment of silicone molecules. Its membrane was supposed to be impermeable between the silicone layers. The studies by Cronin and Gerow^1^ were the ones responsible for the development and dissemination of the use of silicone gel breast implant.

According to Lodovici^10^, the medical grade silicone used for manufacturing the implants meets Scales’ requirements and, therefore, is the inclusion material that is most often used in augmentation mammoplasty. Currently, the implants have, in their interior, silicone with a higher degree of cohesiveness, granting greater safety to the patient in case of possible rupture of the implant; however, the coating of these implants is still under study, since capsular contracture is one of the main late complications of this type of surgery^3^.

Smooth breast implants fell into disuse for many years because capsular contracture occurred five times more in these patients when compared to textured implants^11-13^. Alternatively, polyurethane-coated implants have been used since the 1970s, but with some studies showing possible negative effects of this type of coating^14^.

These effects were already being questioned since 1991 by the Food and Drug Administration (FDA), which interrogated manufacturers regarding the implants’ quality, as complications such as ruptures, contractures, breast cancer risk and others were very common. They also raised suspicion regarding 2,4-toluenediamine (TDA), which results from the degradation of polyurethane. The implants were withdrawn from the U.S. market in the following year. The good news came in 1995, when FDA allowed the use of polyurethane, judging the risk of cancer to be minimal. In 2000, saline implants were allowed for commercialization. Today, FDA grants an approval letter for silicone gel implants^3,14^.

Studies by Wagenführ Jr^7^ and Balderrama^8^, presented the silicone foam as a new type of envelope/coating for breast implants. These experimental studies showed the biocompatibility of this new material, with lower inflammatory response to foreign bodies and without the biodegradable components present in polyurethane. They also show a lower index of capsular contracture and greater integration of the implants into the adjacent tissues, which may lead to a lower rate of complications associated with augmentation mammoplasties. And it was in search of these possible findings that the present clinical study began.

The qualitative variables along the different postoperative periods are described by FDA as possible findings and complications after augmentation mammoplasty; the inframammary scars, which had very satisfactory aesthetic quality, especially when well positioned on the sulcus, were evaluated. Keloid was found in only one patient (Group A) and has no significant relevance (although more common in Afro-descendants and Asians, the keloid patient was of Caucasian origin); the treatment was performed with serial infiltrations of triamcinolone (20 mg/ml). The debridement of the borders, sometimes necessary due to the friction caused by the introduction of the implants, especially those foam coated (and also by the implants of greater volume) could lead to a greater tension in the suture, being able to cause poor scars. This fact is of relevance in the Brazilian population^15^, which has the habit of wearing intimate and bath items of small sizes; but such differences were not observed with significance in the present study.

The presence of capsular contracture also showed no statistical difference. This is one of the major issues assessed throughout the study; and because it is one of the late complications, perhaps a longer follow-up period may show different results. The capsular contracture index presented (maximum 6.3%) is in agreement with most studies in the literature, since this complication is reported in 2.8 to 26.9% of cases of breast augmentation^12,13,15,16^. One of these patients (group B), with bilateral hardening, completed 3 years of follow-up, reporting worsening of breast consistency, with visible (although still small) deformity and sporadic pain, being classified as Baker 4; she has been scheduled for surgical retreatment. The other patient with contracture completed 2 years of follow-up and is asymptomatic.

Regarding the stretch marks, a correlation between the patients’ complaints, the physical examination and the comparison between the photos of the different postoperative periods was held because the gradual improvement of the appearance of the stretch marks throughout the study was noticed, making it difficult to confirm whether they were stretch marks caused before or after the surgery. When present, the stretch marks showed to be more prevalent bilaterally, hydration with oils and nocturnal use of retinoids having been prescribed, leading to a significant improvement of appearance. The comparison of this item received special attention due to the use of slightly larger volume^16^ implants in group A (silicone foam) and not the type of coating, since there would be little influence in the presence of stretch marks; thus, larger volumes could cause greater stretching of the skin^15^, favoring stretch marks. However, there was also no statistical difference between groups.

When evaluating the patients’ degree of satisfaction, there was a higher rate of very satisfied patients in group A (silicone foam), statistically significant for the 1-year follow-up period (p=0.036). In previous periods, the patient who was not satisfied belonged to group B and desired larger implants (mainly after the edema’s regression), her satisfaction level in relation to the result having been classified as “indifferent”. The dissatisfied ones were the two patients who needed surgical retreatment (one for enlargement of the mammary store and improvement of the implants’ positioning and another for fixation of the inframammary sulcus, since the implants had “slipped” towards the abdomen, mainly the left one). So, after one year of surgery, patients who received implants coated with silicone foam showed a higher satisfaction rate than the control group (p<0.05).

In relation to breast ptosis over time, the absence of differences between the groups can also be explained by the short follow-up period for this variable, considering that most patients are young, have no flabby skin and are not pregnant. The volume of the implants presented a significant difference when measured volume in ml (with larger volumes of silicone foam implants), but it is known from the median that the difference was only one size above the manufacturer’s grid, which for plastic surgeons, implies few clinical differences. Thus, the larger volume was not responsible for greater ptosis or presence of a greater amount of stretch marks in group A (without significant difference for these two variables, when comparing the groups).

The patients had body mass index (BMI) less than 25 kg/m^2^; thus, a possible postoperative observation would be the presence of rippling in very thin patients. However, this finding was not significant when comparing the groups at different postoperative follow-up periods.

No infection in the surgical wound or seroma, breast hematoma or rupture of implants was noted in the patients studied. These postoperative findings are consistent with the literature on augmentation mammoplasty^15-19^. The number of complications shown by the patients in this study was lower than the average described in the literature^10,14-18^.

Recent scientific reports have suggested a possible association between anaplastic large cell lymphoma (ALCL) and breast implants, mainly macrotextured ones also found in the literature as BIA-ALCL (Breast Implant-Associated Anaplastic Large Cell Lymphoma). Although there is work published by Jong^20^in 2008 on the subject (with 2 patients), the first reports to the FDA were in 2011 , but only in 2016 the World Health Organization recognized such T-cell lymphoma (non-HodgKin) associated with textured breast implants. By September 2018, the FDA had received a total of 660 patient reports with BIA-ALCL associated with the implants, including the death of nine patients. However, some cases were reported in duplicity, thus leaving 457 cases reported. The current literature has reported several estimates that the BIA-ALCL can develop in 1 of 2,832 to 30,000 women with textured breast implants. The exact number of cases remains hard to determine due to significant limitations in the worldwide reporting and lack of accurate data on the use of breast implants^21-26^

ALCL is usually found close to the implant itself and/or contained within the fibrous capsule. The most common symptom is seroma (60-90% of the patients)^27^, other symptoms such as pain, nodules, swelling or asymmetry are the main findings.

After correct diagnosis, ALCL is usually treated with surgery to remove the implant and adjacent scar tissue, accompanied by periodic oncological observation^23^; chemotherapy and/or radiation therapy are indicated in specific cases. Therefore, it is known that women with breast implants may be at an increased risk of developing ALCL, although the current literature indicates that the risk is extremely low, with no need for a change in routine medical care and follow-up of the patients, being asymptomatic. Considering that this study began in 2014, there was no initial intention to investigate the BIA-ALCL, since there was not an expressive number of reported cases; thus, with patient follow-up we may have in the future contributions from this work to a possible association (or not) of this lymphoma with silicone implants (although no case reported with this type of silicone foam implants).

In the case of the silicone foam implant, its coating is made up of 100% dimethylsiloxane elastomer, which has an adhesion with surrounding tissues, leaving no friction and possible seroma formation, possibly generating a low ALCL risk (unlike other macrotextures). In these way, as the silicone implant adherence is definitive, there is a permanent bond between the implant and the capsule. However, there are some theories that argue that a larger surface area in textured implants can increase bacterial adhesion and biofilm formation, leading to a greater risk of chronic antigen-guided inflammation (response predominantly via Th1 and Th17 lymphocytes)^24,27^whereas regulatory T cells in the capsules are defective in suppressing these intracapsular T cells^6^.

Still, recent studies^28-30^ estimate the risk of developing connective tissue diseases after the inclusion of silicone implants can vary from 0.8% to 25% of patients, in which some authors point out that these numbers may correspond to a much higher risk for patients who received implants when compared to the general population. However, some studies admitted only patients who met the criteria for known autoimmune diseases, hence the difference between the data presented in the literature. Therefore, some studies do not consider patients with less specific manifestations, such as arthralgia, myalgia or even neurological manifestations, but who do not fit into any known condition. Still, even with the improvement in the quality of the coatings of the implants, some authors argue that the autoimmune diseases related to breast implants have not changed in the last 30 years, and there should be a more careful use of these materials, to guarantee patient’s safety^31^.

To date, no patient has had silicone implant-related autoimmune disease (including adjuvant-induced autoimmune / inflammatory syndrome - ASIA). All women included in the study remain in follow-up and may contribute to new articles on the subject in the future.

## Conclusion

Silicone foam envelope implants proved to be as reliable as textured silicone envelope implants, making them an option for augmentation mammoplasty.
